# Guiding nanomaterials to tumors for breast cancer precision medicine: from tumor-targeting small-molecule discovery to targeted nanodrug delivery

**DOI:** 10.1038/am.2017.196

**Published:** 2017-12-08

**Authors:** Xuewei Qu, Penghe Qiu, Ye Zhu, Mingying Yang, Chuanbin Mao

**Affiliations:** 1 Stephenson Life Sciences Research Center, Department of Chemistry and Biochemistry, University of Oklahoma, Norman, OK, USA; 2 Institute of Applied Bioresource Research, College of Animal Science, Zhejiang University, Hangzhou, Zhejiang, China; 3 School of Materials Science and Engineering, Zhejiang University, Hangzhou, Zhejiang, China

## Abstract

Precision medicine emphasizes patient-specific formulation for treatment of diseases, especially cancer. However, in targeted cancer treatment, because the expression level of tumor receptors in each patient varies even for the same type of cancer, the ligand/receptor-mediated approach does not seem promising for precision medicine. In this work, we demonstrated our strategy of using a phage display technique for breast cancer precision medicine. Using *in vivo* biopanning, we first selected an MCF-7 breast tumor-targeting peptide, then tested the effectiveness of the as-selected peptide in tumor homing and finally conjugated the peptide to a model photothermal drug, namely, gold nanorods, to achieve enhanced cancer killing efficacy. The peptides identified by the phage display technique can guide the drug to the tumors without the need to know the exact receptors on the tumor. This approach requires significantly less effort to explore patient-specific targeting molecules for precision medicine.

## INTRODUCTION

**Figure 1:**
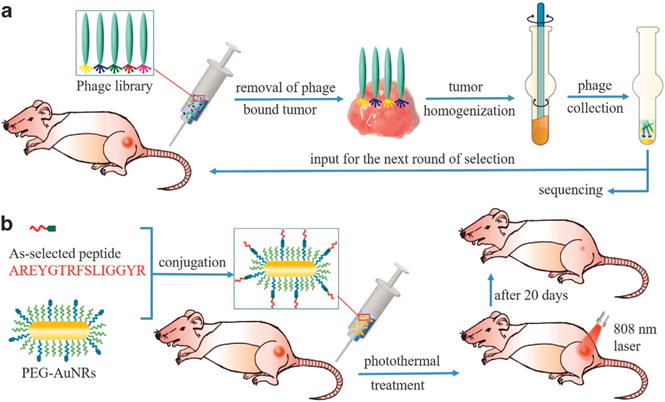
Schematic description of phage display-based breast cancer precision medicine. (**a**) Selection of patient-specific breast cancer-targeting peptides through *in vivo* phage display. A phage library was first injected through the tail vein into the tumor-bearing mice and allowed to circulate for 24 h. Tumors with preferentially bound phages were removed from mice and homogenized. Phages were collected by centrifugation, amplified and used as input for the next round of *in vivo* selection. (**b**) Coupling of the as-selected peptide with anticancer nanomedicines (AuNRs) to enhance their accumulation inside tumors to achieve highly efficient cancer treatment by photothermal therapy.

In the most recent decade, targeted drug delivery has been studied intensively for cancer treatment.^1–4^ This method involves the use of tumor-recognizing molecules in the drug formulation to enhance drug accumulation in cancerous tissue. This strategy can result in significantly enhanced cancer treatment efficacy, decreased side effects and reduced drug use. Until now, different types of molecules with tumor affinity, such as antibodies,^5–9^ folic acid/folate^10–15^ and hyaluronic acid,^16–19^ have been reported and applied for targeted drug delivery. These molecules interact preferentially with certain types of proteins that are overexpressed or specifically expressed at the tumor sites. This type of tumor-targeting approach requires unambiguous previous knowledge of the corresponding tumor receptors that are paired with these targeting molecules.^20–23^ However, such a strategy neglects patient-specific tumor differences for the same type of cancer. Ideally, a molecule that targets patient-specific tumors should be identified and integrated with cancer therapeutics to achieve customized cancer therapy. This is the goal of precision medicine in the context of cancer therapy. Precision medicine is a new emerging research area that aims to deliver personalized drug formulations or treatment plans for individual patients.^24,25^ It is expected that with the use of patient-specific solutions, cancer treatment can be significantly more effective. As each type of cancer differs in many ways in different patients, especially on the molecular level, the tumor receptor-based targeted delivery strategy might not work as well for precision medicine, because the expression level of the receptors might vary dramatically among patients. This is also one of the reasons why current cancer therapeutics show low survival rates. In addition, for unknown types of tumors or new tumor variants in individual patients, it is difficult in practice to explore the specific ligand–receptor pairs if the patient-specific receptors have not been identified. In this work, we show that without considering the unique receptors of breast tumors, we are able to discover novel peptides capable of preferentially recognizing the tumors via the phage display technique. This approach is possible because identification of targeting peptides by phage display in principle does not require prior knowledge of the target and thus peptides with affinities to unknown types of tumors can be selected in a fast and cost-effective manner, allowing us to identify patient-specific tumor-homing peptides. We intend to demonstrate a new strategy for breast cancer precision medicine using MCF-7 breast cancer-bearing mice as the model in which a tumor-homing peptide customized to a specific patient is first identified without the need to know what receptors this peptide recognizes and is subsequently linked with cancer nanomedicine (using photothermal gold nanorods or AuNRs as a model drug) to selectively inhibit the tumor (Figure 1). For the first time, we demonstrate such a strategy by performing the entire procedure from the discovery of tumor-homing peptides to the eventual validation of the peptide in targeted breast cancer therapy using the same animal model in the same lab.

## MATERIALS AND METHODS

### Cell line and MCF-7 tumor xenograft

Human MCF-7 breast cancer cells (ATCC, Manassas, VA, USA) were cultured in Eagle’s minimal essential medium (ATCC) with 10% fetal bovine serum (Gibco, Thermo Fisher Scientific, Waltham, MA, USA). To generate tumors in mice, 2.5–3 × 10^6^ cells in 0.1 ml of saline were subcutaneously injected into the right flank of each mouse (Athymic Nude-Foxn1^nu^, 3–5 weeks, female, Harlan Lab, Indianapolis, IN, USA). All tests of the *in vivo* phage display and photothermal therapy began when the tumor reached 0.5–0.8 cm in diameter.

### *In vivo* phage display

The f3–15mer phage library (aka fUSE/15mer; GenBank Accession AF246445), which displays a 15-mer peptide on all five copies of pIII coat proteins at the N-terminus on fd phages, was a gift from Dr George Smith at the University of Missouri. At least 1 × 10^12^ transducing units of the f3-15mer phage library were intravenously injected into each tumor-bearing mouse through the tail vein and allowed to circulate for 1 h before euthanasia by CO_2_ asphyxiation. Subsequently, 20 ml of phosphate-buffered saline buffer was used to wash the unassociated phages by heart perfusion. The tumor-recognized phages were purified from the tumor homogenate and amplified for use as new input phages for the next round of selection. The tumor-homing peptide sequences in every round of selection were identified by phage genome sequencing from the third to fifth round.

### *In vivo* fluorescence imaging

All peptides with biotin labeled at the C-terminal for conjugation with streptavidin were purchased from United BioSystems (Herndon, VA, USA). A linker of GGG was added to the C-terminal during peptide synthesis for each peptide. The streptavidin proteins, which were tagged with a fluorescent dye (Cy5) at a dye/protein ratio of 6.5, were purchased from USBiological (Salem, MA, USA). To conduct conjugation, 16 μl of 1 mg ml^−1^ streptavidin was added to 800 μl of 6.0 μM peptide saline solution and the mixture was incubated at room temperature for 3 h. Free unconjugated peptide was removed using Slide-A-Lyzer Dialysis Cassettes (10 000 MWCO, Thermo Fisher Scientific, Waltham, MA, USA) according to the protocols supplied with the kit. The Cy5-peptide conjugates were stored at 4 °C and used within 24 h after preparation. For *in vivo* imaging, 5 nmol of the as-prepared Cy5-peptides conjugates dissolved in 100 μl saline was injected through tail vein of each tumor model. All mice were given anesthesia consisting of a 4% isoflurane/oxygen mixture and scanned under the *In vivo* Xtreme Imaging System (Carestream, Inc., Rochester, NY, USA) 24 h after injection.

### Synthesis of gold nanorods

AuNRs were synthesized using a seed-mediated process. The seed solution was prepared by adding 600 μl of NaBH_4_ (100 mM) into 10 ml of a mixture solution of 0.25 mM HAuCl_4_ and 0.1 M cetyl trimethylammonium bromide (CTAB) under vigorous stirring. The seed solution was used within 2 h after preparation. A total of 500 ml of AuNRs solution was prepared each time. In brief, to a 495 ml growth solution containing 18.2 g CTAB, 25 ml of 10 mM HAuCl4, 5 ml of 10 mM AgNO3, and 10 ml of 1.0 M HCl, 4 ml of 0.1 M ascorbic acid and 1.2 ml of the seed solution were injected subsequently. The solution was mixed by gentle shaking during each addition. Afterwards, the solution was left untouched for overnight to allow AuNRs growth. The longitudinal absorption peak of the resultant AuNRs was located around 808 nm.

### Preparation of peptide-conjugated tumor-homing AuNRs

The peptides used in AuNRs conjugation were synthesized with three glycine amino acids (GGG) and four additional lysine amino acids (KKKK) at the C-terminal. It is expected that the majority of the conjugation reaction occurs through the amine groups at the C-terminal, leaving the –NH_2_ at the N-end free. Conjugation of peptides onto AuNRs can be completed using a three-step surface modification. First, 100 ml of the as-prepared AuNRs was centrifuged twice at 10 000 rpm for 10 min, redispersed into 50 ml of 0.1 mM thiol functionalized polyethylene glycol (HS-PEG, MW of 2 kDa) aqueous solution and placed overnight, to allow the CTAB molecules on the AuNRs surface to be replaced with HS-PEG molecules. The AuNRs were then centrifuged once and mixed with 50 ml of 0.1 mM HS-PEG-COOH for a few hours to achieve partial ligand exchange of HS-PEG by HS-PEG-COOH. Subsequently, the nanoparticles were subjected to another round of centrifugation and redispersed into 10 ml of pH 6.0 MES buffer. The peptide conjugation was initiated by activation of –COOH with 1-ethyl-3-(3-dimethylaminopropyl) carbodiimide (EDC, 4 mg) and *N*-hydroxysuccinimide (NHS, 6 mg) at room temperature for 15 min, followed by centrifugation at 4 °C for 10 min. Thereafter, the nanoparticle pellet was immediately resuspended into 2 ml of 1 mM peptide/ saline solution and left on a rocker shaker for 2 h under room temperature. The resultant peptide-conjugated tumor-homing AuNRs had a concentration of ~ 5 mg ml^−1^ and 200 μl of the solution containing ~ 1 mg of AuNRs was used in each mouse for intravenous injection.

### Quantification of peptides on AuNRs

The targeting peptide with a Cy5 tag on the N-terminal was used in conjugation with AuNRs-HS-PEG-COOH through EDC/NHS chemistry. After repeated washing to remove excess peptides, the functionalized AuNRs (equivalent to 100 ml of as-synthesized AuNRs) were redispersed into 2 ml of water. Then, 0.5 ml of 0.1 M potassium cyanide was added to dissolve the AuNRs. Hence, the surface ligands on the AuNRs, including the Cy5-tagged peptides, were simultaneously released as free molecules. The concentration of the peptides was measured via the fluorescence intensity of the Cy5 tag at excitation and emission wavelengths of 650 and 670 nm, respectively. Assuming the AuNRs were round-ended cylinders of 40 nm in length and 10 nm in diameter, the average number of peptides on individual AuNRs was calculated to be 141, corresponding to a surface density of ~ 0.09 peptide per nm^2^.

### Quantification of AuNRs tissue distribution through inductively coupled plasma atomic emission spectroscopy (ICP-AES)

Peptide 1 or control peptide-conjugated AuNRs (200 μl, 5 mg ml^−1^) were intravenously injected into each tumor-bearing mouse (5 mice per group) through the tail vein and circulated for 1 to 5 days before killing. The tumors and other organs, including the liver, kidney, heart and lung, were excised after heart perfusion and completely dried in a freeze-drying system. Peripheral blood was collected before heart perfusion as well. Subsequently, all tissues were weighed and digested completely by freshly prepared aqua regia. Finally, the solution was diluted with a large amount of water, filtered and used for ICP-AES quantification.

To track the gold concentration in peripheral blood, 20 μl of blood was collected from the suborbital space of five anesthesia-treated mice each time at 3 min, 20 min, 1, 4, 7, 12, 24 and 48 h after injection of either peptide-conjugated AuNRs or PEG-modified AuNRs. The gold was digested and quantified following the same procedures as described above.

### Photothermal treatment of MCF-7 tumor

A total of 20 tumor-bearing mice were randomly separated into four groups. Mice in the four groups were injected intravenously with target peptide-AuNRs, control peptide-AuNRs, PEG-AuNRs or saline. The amount of AuNRs was 1 mg per mouse. Twenty-four hours after injection and under general anesthesia, all mice were irradiated at the tumor site with an 808 nm laser for 5 min at a power density of 2.25 W cm^−2^. The laser spot size was ~ 1 cm in diameter. Thermal imaging was conducted using an infrared camera (ICI 7320, Infrared Cameras, Inc., Beaumont, TX, USA) to track the temperature increase inside the tumors during the photothermal treatment. After laser irradiation, the mice were housed for another 20 days, after which the tumor dimensions were measured and the tumors were removed from the mice and weighed. The tumor volume was calculated using the following equation: Volume = length × width^2^/2.

### Hematoxylin and eosin staining

For hematoxylin and eosin (HE) staining, tumors were excised from mice 3 h after laser treatment, placed immediately into 10% fresh formalin solution and soaked for 24 h. The fixed tumors were embedded in a paraffin block according to standard procedures. Thereafter, the tumors were cut into 5-μm-thick sections and mounted onto glass slides. Before imaging, the slices were deparaffinated and subsequently stained with HE staining solutions. The imaging was performed on an Eclipse Ti microscopy system (Nikon Instruments, Inc., Melville, NY, USA).

## RESULTS AND DISCUSSION

In brief, *in vivo* phage display was performed in several rounds of affinity testing against MCF-7 tumors *in vivo*. In the first round, a phage library, which contains billions of phage clones with each displaying a unique 15-mer peptide at the five copies of minor coat protein (pIII) of the phage, was injected intravenously into tumor-bearing mice. After an hour, the mice were sacrificed, and the tumors were excised and homogenized. Tumor-associated phages (output phages) were extracted from tumor homogenate and used as an input for the next round of selection. Similarly, in the remaining rounds of selections, output from the previous round was used as an input for the next round and injected intravenously into a new tumor-bearing mouse. In this work, a total of five rounds of selection were conducted. It is expected that phages appearing in the output of subsequent rounds should have higher tumor affinity. Hence, we randomly isolated selected output phage clones in rounds 3, 4 and 5. As the DNA inside the phage genetically encodes the peptides displayed on the surface of the phage, the DNA of the tumor-associated phage clones was sequenced to identify the sequences of 15-mer peptides (Table 1 and Supplementary Tables S1). A total of 246 colonies were identified from 3 to 5 rounds of *in vivo* selection and the top 12 identified peptides with a number of repeats no <2 after the fifth round are listed in Table 1. Our subsequent studies primarily focused on the top five peptides with the highest occurrence frequencies, namely, AREYGTRFSLIGGYR (peptide 1), PKAFQYGGRAVGGLW (peptide 2), PVRYGFSGPRLAELW (peptide 3), RNVPPIFKEVYWIAQ (peptide 4) and RTLIRMGTGAHAFAV (peptide 5), because they are more likely to have higher affinity to MCF-7 tumors than the other peptides. Among these peptides, peptide 1 has the highest occurrence frequency, indicating that peptide 1 has the highest tumor affinity.

We conducted *in vivo* experiments to test the excellence of the selected peptides in tumor targeting. First, we used the *in vivo* imaging system to verify peptide accumulation at tumor sites. Second, we conjugated the peptides to gold nanorods, a well-known photothermal reagent,^26^ to investigate the effectiveness of the selected peptides in enhancing the targeted delivery of drugs to cancerous tissues.

**Figure 2:**
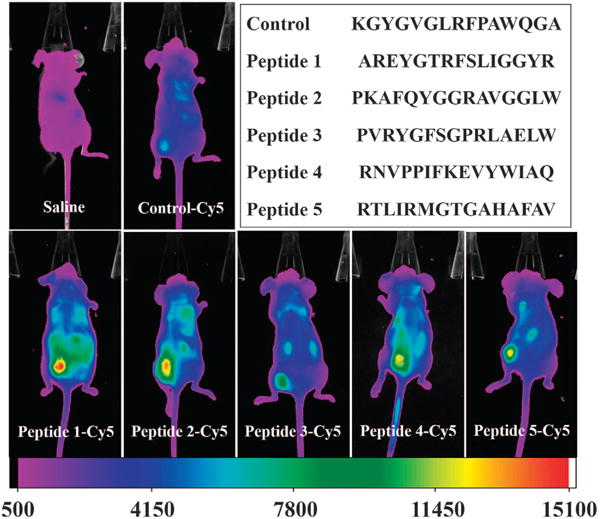
*In vivo* imaging for detection of selective accumulation of tumor-homing peptides at the MCF-7 tumors. Peptides were first labeled with biotin at the C-terminus and then conjugated to Cy-5-tagged streptavidin. Images were acquired 24 h after intravenous injection of 5 nmol of peptides (100 μl in saline). All images were acquired under the same parameters and thus the intensity difference represents the actual difference in peptide accumulation at the tumor sites.

For *in vivo* imaging, the five peptides were first biotinylated (see Supplementary Figure S1 for detailed structure), then conjugated to streptavidin with a Cy5 fluorescent tag and finally injected into mice through the tail vein. *In vivo* imaging was performed 24 h later. For comparison, a random peptide, KGYGVGLRFPAWQGA, was used as a control peptide. The amount of peptides accumulated at the MCF-7 tumors is judged by the fluorescence intensity, and the stronger fluorescence indicates greater peptide accumulation. As shown in Figure 2, all of the five selected peptides exhibited significantly higher tumor accumulation than the control peptide. Among these peptides, peptide 1 showed the strongest fluorescence intensity, suggesting that it has the best tumor-homing capability. In addition, peptide 2 presented slightly lower fluorescence intensity than peptide 1, which is consistent with the fact that peptide 1 has slightly higher occurrence frequency than peptide 2 (Table 1). Thus, peptide 1 was chosen for the following targeted drug delivery tests.

**Figure 3:**
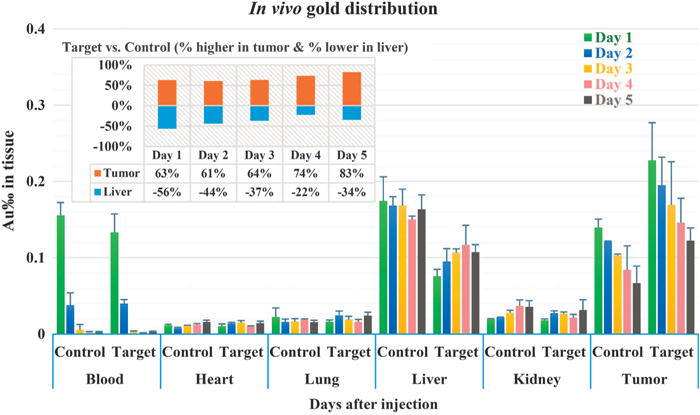
Organ distribution of AuNRs. Mice were killed 1, 2, 3, 4 and 5 days after intravenous injection of 1 mg of AuNRs. The target group was the group injected with peptide-1-conjugated AuNRs, whereas the control group was injected with control-peptide-conjugated AuNRs. Gold concentration was calculated as mass permillage of Au (Au‰) in the corresponding tissue. The inset chart shows the percentage increase (in tumor, represented as positive values) or decrease (in liver, represented as negative values) of gold content in the target group compared with the control group on different days. The data indicate that conjugation of our selected peptide 1 onto AuNRs can effectively increase their uptake by tumors but decrease their uptake by livers.

The peptide-assisted *in vivo* drug delivery was tested by quantification of nanoparticle tissue distribution as well as the effectiveness of photothermal treatment on MCF-7 tumor-bearing mice. To conduct the test, peptide 1 was first conjugated with AuNRs at the C-terminal through a well-developed method in which the as-synthesized CTAB-coated AuNRs were first replaced by HS-PEG-COOH, followed by peptide conjugation via 1-ethyl-3-(3-dimethylaminopropyl) carbodiimide/*N*-hydroxysuccinimide chemistry (see Experimental section).^27,28^ The resultant nanoparticles are referred to as peptide-AuNRs. The TEM images and optical spectra of AuNRs (Supplementary Figure S2) show that the multi-step modification did not cause destabilization of the nanoparticles. The hydrodynamic radii (as measured by dynamic light scattering, DLS) of the CTAB-coated AuNRs, PEG-coated AuNRs and peptide-conjugated AuNRs in water are 40.8 ± 2.1 nm, 44.3 ± 2.5 nm and 47.0 ± 2.4 nm, respectively (Supplementary Figure S3a–c), suggesting successful conjugation of peptides onto the AuNRs. The hydrodynamic radius increased only slightly to 47.6 ± 2.7 nm when the peptide-AuNRs were transferred from water to phosphate-buffered saline (Supplementary Figure S3d), which demonstrates the high stability of AuNRs in physiological conditions. For quantitative studies, 1 mg of peptide-AuNRs (200 μl, 5 mg ml^−1^) per mouse was used in intravenous injection. We first monitored the *in vivo* blood circulation of AuNRs for 48 h. We discovered that the half-life (*t*_1/2_) of the target-AuNRs in the blood is ~ 16 h (Supplementary Figure S4) and the value for the control peptide-conjugated AuNRs (Control-AuNRs) is approximately 18 h, and both are comparable to the previously reported t_1/2_ of PEG-coated AuNRs.^29^ For the *in vivo* tissue distribution study, the nanoparticles were allowed to circulate for five different periods of time (1, 2, 3, 4 and 5 days). At the end of each time point, the mice were sacrificed, and the organs were freeze dried, weighed, and digested. Finally, the concentration of gold in each organ was quantified by ICP-AES. The control-AuNRs were used as a control. In the presence of the tumor-homing peptide, the tumor uptake of AuNRs after 1 day of circulation was 63% higher than that of the case with the control peptide, and it remained 61–83% higher during the entire period of 5-day monitoring (Figure 3). This result confirms that peptide 1 selected from the *in vivo* phage display can result in significantly enhanced drug delivery to the MCF-7 tumors. The distribution of the target-AuNRs in other healthy organs is either less than or comparable to that of the control-AuNRs. It is worth noting that the gold content in the liver in the target group is 56% lower than that in the control group on day 1 and remained 22–44% lower in the following 4 days, suggesting that modification of AuNRs with the tumor-targeting peptides can effectively reduce non-specific AuNRs uptake by the liver.

**Figure 4:**
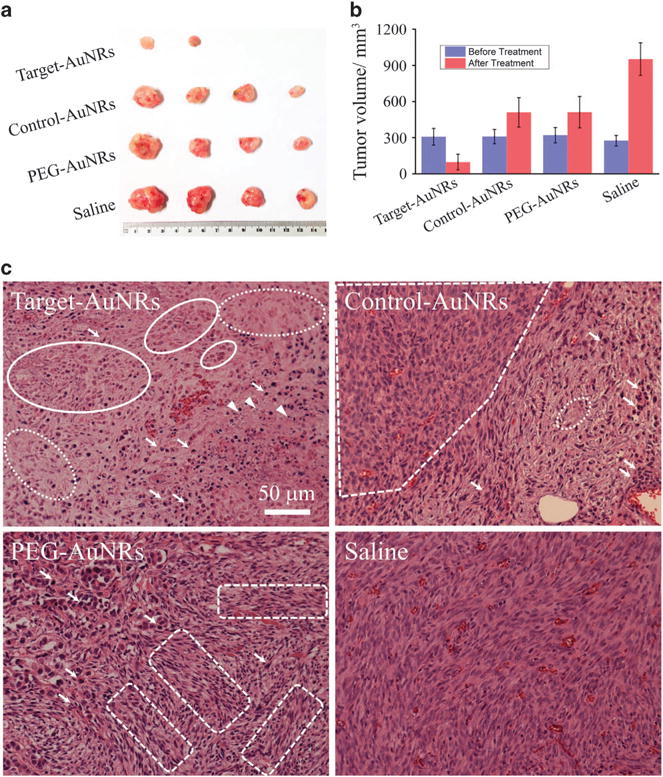
Photothermal treatment of MCF-7 tumors. (**a**) Photographs of typical tumors removed from mice 20 days after photothermal therapy (for the target-AuNRs group, two of the tumors disappeared completely); (**b**) average tumor volume before and 20 days after the treatment; (**c**) images of HE stained sections of tumors obtained 3 h after 808 nm laser treatment. Target-AuNRs, AuNRs modified with peptide 1; PEG-AuNRs, PEG-modified AuNRs; Control-AuNRs, AuNRs modified with a control peptide; Saline, saline only and without AuNRs. Compared with the normal nucleus of cancer cells in the control tumor, contiguous cell necrosis is determined by the extensive pale eosinophilic cytoplasm (typically highlighted by dashed ovals), as well as karyolysis and nuclear swelling (typically highlighted by solid ovals). Cell apoptosis with the presence of nuclear pyknosis, hepereosinophilic cytoplasm (typically highlighted by arrows) and apoptotic bodies (typically highlighted by arrow heads) is marked. The normal tumor tissue area in each AuNRs treated group is also circled with angled dashed frames.

Photothermal treatment was conducted 24 h after injection because the concentration of peptide-AuNRs in tumors is maximized at this time point, according to the ICP-AES based biodistribution study. During the treatment, MCF-7 tumors were irradiated directly with an 808 nm laser at a power density of 2.25 W cm^−2^ for 5 min. Thermal imaging (Supplementary Figure S5) shows that the temperature increase inside the tumors is much higher in the target-AuNRs group than in other groups, whereas the tumor temperatures in the PEG-AuNRs and the control-AuNRs groups are generally comparable. The average temperatures measured at the end of the 5 min treatment period are 31.5 ± 2.2 °C (saline), 45.6 ± 5.6 °C (PEG-AuNRs), 46.8 ± 5.4 °C (control-AuNRs) and 56.2 ± 7.2 °C (target-AuNRs). Figure 4 shows photographs of the tumors and the corresponding average tumor volumes in each group 20 days after photothermal therapy. It is clear that the size and volume of the tumors in the targeting group (AuNRs functionalized with peptide 1) are significantly smaller than those in the other groups. In fact, two of the tumors in this group were so severely destroyed that it was difficult to collect any tumor tissue even after 20 days following the treatment. The development of tumors treated by non-targeting AuNRs modified with PEG and control peptide was also retarded, although not as significantly as that in the targeting group because even without the assistance of targeting peptide, AuNRs can still be delivered to tumors through the passive targeting mechanism.

HE staining further illustrates the death of tumor cells as a result of photothermal treatment (Figure 4c). Tumors used in this study were collected from mice 3 h after the laser treatment. For control tumors without AuNRs (saline group), both apoptosis and necrosis (the two typical cell death mechanisms) are barely found in the tumor cells, suggesting that the laser power density used in the photothermal treatment was safe for normal tissues. However, for tumors in the target group (target-AuNRs), the classic necrosis characteristics, including karyolysis, nuclear swelling and extensive pale eosinophilic cytoplasm, are all observed, indicating contiguous cell necrosis upon acute injury caused by photothermal treatment. In addition, apoptosis characteristics such as nuclear pyknosis, hypereosinophilic cytoplasm and apoptotic bodies were also observed, which are indications of programmable cell death following the laser treatment. In contrast, such damages in the other two AuNRs groups (PEG-AuNRs and control-AuNRs) were not as significant (see Supplementary Figure S6 for lower magnification images). Overall, our photothermal investigation is in accordance with the study of organ distribution of AuNRs by ICP-AES in that the *in vivo* selected targeting peptides, when conjugated onto AuNRs, can indeed result in enhanced delivery and accumulation of AuNRs at the tumor sites, which further leads to remarkably improved efficiency in the photothermal cancer therapy.

Phage display can be performed both *in vitro* and *in vivo*. In the *in vitro* phage display approach, the phage library is allowed to interact with cancer cells in the culture media, whereas in the *in vivo* phage display approach, it is intravenously injected into animals and circulated in the blood stream to enable the discovery of phages that home to tumors. In principle, *in vivo* selection against tumors should be more target-specific and more effective for clinical applications because the peptides were selected under conditions similar to the physiological environment. Thus far, no research group has studied the discovery of tumor-targeting peptides using *in vivo* phage display and subsequently tested the use of such peptides in *in vivo* homing and destruction of tumors, all using the same animal models and in the same lab.^30–33^ Most of the reported studies were based on peptides reported previously by others rather than selected through exactly the same tumor model in the same work, and thus, these peptides cannot be considered patient specific. Hence, these works did not meet the requirement of precision medicine. In particular, for MCF-7 breast cancer, such studies were reported exclusively based on peptides selected through *in vitro* phage display.^34–37^ In addition, although we demonstrated the concept of phage display-based breast cancer precision medicine using only photothermal treatment, the same procedures can be readily applied to other cancer therapeutics such as chemotherapy and gene and photodynamic therapies.

## CONCLUSION

In summary, we have demonstrated a new strategy of breast cancer precision medicine using the MCF-7 breast cancer mouse model. In this strategy, a patient-specific tumor-targeting peptide was first identified through *in vivo* phage display. Our *in vivo* imaging study showed that these peptides can actively target MCF-7 tumors. By conjugating the as-selected peptides with the model drug of AuNRs, enhanced accumulation of the nanomedicine to the tumors was observed by ICP-AES measurement, and this accumulation further leads to improved cancer killing efficiency.

## Supplementary Material

SI

## Figures and Tables

**Table 1:** Summary of peptide sequences and frequencies in the third to fifth rounds of *in vivo* phage display against MCF-7 tumors^a^

Peptide sequences	Count/frequency (%)			
	3rd Rnd (30 cln)	4th Rnd (54 cln)	5th Rnd (162 cln)	Sum (246 cln)
AREYGTRFSLIGGYR	1/3.33	3/5.56	7/4.32	11/4.30
PKAFQYGGRAVGGLW	0	3/5.56	7/4.32	10/4.07
PVRYGFSGPRLAELW	0	0	3/1.85	3/1.22
RNVPPIFKEVYWIAQ	0	0	3/1.85	3/1.22
RTLIRMGTGAHAFAV	0	0	3/1.85	3/1.22
DRYLPINGVSMFGVP	0	0	2/1.23	2/0.81
IPVQFSTIDFVAASY	0	0	2/1.23	2/0.81
PGHSLGKLSVLHSFF	0	0	2/1.23	2/0.81
QADGPNSVVRPFTLT	0	0	2/1.23	2/0.81
RSFAYAAAPTSFPWV	0	0	2/1.23	2/0.81
SVGCPVVGTVGYLRCG	0	0	2/1.23	2/0.81
VRMFDYGVPRRAVYGG	0	0	2/1.23	2/0.81

Abbreviations: cln, total phage colonies; Rd, round of biopanning.

a Only peptides with total colonies no <2 are included in this table.

## References

[1] Abeylath SC, Ganta S, Iyer AK, Amiji M (2011). Combinatorial-designed multi-functional polymeric nanosystems for tumor-targeted therapeutic delivery. Acc Chem Res.

[2] Caldorera-Moore ME, Liechty WB, Peppas NA (2011). Responsive theranostic systems: integration of diagnostic imaging agents and responsive controlled release drug delivery carriers. Acc Chem Res.

[3] Shi JJ, Xiao ZY, Kamaly N, Farokhzad OC (2011). Self-assembled targeted nanoparticles: evolution of technologies and bench to bedside translation. Acc Chem Res.

[4] Koo H, Huh MS, Sun IC, Yuk SH, Choi K, Kim K, Kwon IC (2011). *In vivo* targeted delivery of nanoparticles for theranosis. Acc Chem Res.

[5] Scott AM, Wolchok JD, Old LJ (2012). Antibody therapy of cancer. Nat Rev Cancer.

[6] Loo DT, Mather JP (2008). Antibody-based identification of cell surface antigens: targets for cancer therapy. Curr Opin Pharmacol.

[7] Trikha M, Corringham R, Klein B, Rossi JF (2003). Targeted anti-interleukin-6 monoclonal antibody therapy for cancer: a review of the rationale and clinical evidence. Clin Cancer Res.

[8] Schrama D, Reisfeld RA, Becker JC (2006). Antibody targeted drugs as cancer therapeutics. Nat Rev Drug Discov.

[9] Milenic DE, Brady ED, Brechbiel MW (2004). Antibody-targeted radiation cancer therapy. Nat Rev Drug Discov.

[10] Srinivasarao M, Galliford CV, Low PS (2015). Principles in the design of ligand-targeted cancer therapeutics and imaging agents. Nat Rev Drug Discov.

[11] Hilgenbrink AR, Low PS (2005). Folate receptor-mediated drug targeting: from therapeutics to diagnostics. J Pharm Sci.

[12] Paulos CM, Turk MJ, Breur GJ, Low PS (2004). Folate receptor-mediated targeting of therapeutic and imaging agents to activated macrophages in rheumatoid arthritis. Adv Drug Delivery Rev.

[13] Landmark KJ, DiMaggio S, Ward J, Kelly CV, Vogt S, Hong S, Kotlyar A, Myc A, Thomas TP, Penner-Hahn JE, Baker JR, Holl MMB, Orr BG (2008). Synthesis, characterization, and *in vitro* testing of superparamagnetic iron oxide nanoparticles targeted using folic acid-conjugated dendrimers. ACS Nano.

[14] Fan NC, Cheng FY, Ho JAA, Yeh CS (2012). Photocontrolled targeted drug delivery: photocaged biologically active folic acid as a light-responsive tumor-targeting molecule. Angew Chem Int Ed.

[15] Zuber G, Zammut-Italiano L, Dauty E, Behr JP (2003). Targeted gene delivery to cancer cells: directed assembly of nanometric DNA particles coated with folic acid. Angew Chem Int Ed.

[16] Lee YH, Lee H, Kim YB, Kim JY, Hyeon T, Park H, Messersmith PB, Park TG (2008). Bioinspired surface immobilization of hyaluronic acid on monodisperse magnetite nanocrystals for targeted cancer imaging. Adv Mater.

[17] Cho HJ, Yoon HY, Koo H, Ko SH, Shim JS, Lee JH, Kim K, Kwon IC, Kim DD (2011). Self-assembled nanoparticles based on hyaluronic acid-ceramide (HA-CE) and Pluronic (R) for tumor-targeted delivery of docetaxel. Biomaterials.

[18] Jiang TY, Zhang ZH, Zhang YL, Lv HX, Zhou JP, Li CC, Hou LL, Zhang Q (2012). Dual-functional liposomes based on pH-responsive cell-penetrating peptide and hyaluronic acid for tumor-targeted anticancer drug delivery. Biomaterials.

[19] Li JC, He Y, Sun WJ, Luo Y, Cai HD, Pan YQ, Shen MW, Xia JD, Shi XY (2014). Hyaluronic acid-modified hydrothermally synthesized iron oxide nanoparticles for targeted tumor MR imaging. Biomaterials.

[20] Chen C, Ke JY, Zhou XE, Yi W, Brunzelle JS, Li J, Yong EL, Xu HE, Melcher K (2013). Structural basis for molecular recognition of folic acid by folate receptors. Nature.

[21] Mankoff DA, Link JM, Linden HM, Sundararajan L, Krohn KA (2008). Tumor receptor imaging. J Nucl Med.

[22] Zhao XB, Li H, Lee RJ (2008). Targeted drug delivery via folate receptors. Expert Opin Drug Deliv.

[23] Ahrens T, Assmann V, Fieber C, Termeer CC, Herrlich P, Hofmann M, Simon JC (2001). CD44 is the principal mediator of hyaluronic-acid-induced melanoma cell proliferation. J Invest Dermatol.

[24] Collins FS, Varmus H (2015). A new initiative on precision medicine. N Engl J Med.

[25] Schork NJ (2015). Time for one-person trials. Nature.

[26] Huang XH, El-Sayed IH, Qian W, El-Sayed MA (2006). Cancer cell imaging and photothermal therapy in the near-infrared region by using gold nanorods. J Am Chem Soc.

[27] Cho SK, Emoto K, Su LJ, Yang X, Flaig TW, Park W (2014). Functionalized gold nanorods for thermal ablation treatment of bladder cancer. J Biomed Nanotechnol.

[28] Cheng Y, Meyers JD, Agnes RS, Doane TL, Kenney ME, Broome AM, Burda C, Basilion JP (2011). Addressing brain tumors with targeted gold nanoparticles: a new gold standard for hydrophobic drug delivery?. Small.

[29] von Maltzahn G, Park JH, Agrawal A, Bandaru NK, Das SK, Sailor MJ, Bhatia SN (2009). Computationally guided photothermal tumor therapy using long-circulating gold nanorod antennas. Cancer Res.

[30] Arap W, Pasqualini R, Ruoslahti E (1998). Cancer treatment by targeted drug delivery to tumor vasculature in a mouse model. Science.

[31] Matsuo AL, Juliano MA, Figueiredo CR, Batista WL, Tanaka AS, Travassos LR (2011). A new phage-display tumor-homing peptide fused to antiangiogenic peptide generates a novel bioactive molecule with antimelanoma activity. Mol Cancer Res.

[32] Du B, Han HH, Wang ZQ, Kuang LS, Wang L, Yu LP, Wu MA, Zhou ZL, Qian M (2010). Targeted drug delivery to hepatocarcinoma *in vivo* by phage-displayed specific binding peptide. Mol Cancer Res.

[33] Newton JR, Kelly KA, Mahmood U, Weissleder R, Deutscher SL (2006). *In vivo* selection of phage for the optical Imaging of PC-3 human prostate carcinoma in mice. Neoplasia.

[34] Wang T, Hartner WC, Gillespie JW, Praveen KP, Yang SH, Mei LA, Petrenko VA, Torchilin VP (2014). Enhanced tumor delivery and antitumor activity *in vivo* of liposomal doxorubicin modified with MCF-7-specific phage fusion protein. Nanomedicine.

[35] Wang T, D’Souza GGM, Bedi D, Fagbohun OA, Potturi LP, Papahadjopoulos-Sternberg B, Petrenko VA, Torchilin VP (2010). Enhanced binding and killing of target tumor cells by drug-loaded liposomes modified with tumor-specific phage fusion coat protein. Nanomed.

[36] Wang T, Petrenko VA, Torchilin VP (2010). Paclitaxel-loaded polymeric micelles modified with MCF-7 cell-specific phage protein: enhanced binding to target cancer cells and increased cytotoxicity. Mol Pharm.

[37] Wang T, Yang SH, Petrenko VA, Torchilin VP (2010). Cytoplasmic delivery of liposomes into MCF-7 breast cancer cells mediated by cell-specific phage fusion coat protein. Mol Pharm.

