# Supplementary material for: Guiding nanomaterials to tumors for breast cancer precision medicine: from tumor-targeting small-molecule discovery to targeted nanodrug delivery
Source: NPG Asia Mater. Author manuscript; Available in PMC 2018 Apr 13. (PMC5898397; doi:10.1038/am.2017.196)
Supplement: SI [file NIHMS931396-supplement-SI.docx]

**Supporting information**

Toward Breast Cancer Precision Medicine: From Tumor-Targeting Small Molecule Discovery to Targeted Drug Delivery to Breast Tumor

Xuewei Qu^1,4^, Penghe Qiu^1,4^, Ye Zhu^1^, Mingying Yang^2*^, and Chuanbin Mao^1,3*^

^1^Stephenson Life Sciences Research Center, Department of Chemistry and Biochemistry, University of Oklahoma, 101, Stephenson Parkway, Norman, OK, 73019, USA; ^2^Institute of Applied Bioresource Research, College of Animal Science, Zhejiang University, Yuhangtang Road 866, Hangzhou, 310058, China; ^3^School of Materials Science and Engineering, Zhejiang University, Hangzhou, Zhejiang 31002, China

^4^These authors contributed equally to this work.

**Table S1.** 30 sequences were randomly picked and identified from the 3^rd^ round of *in vivo* biopanning.

| Sequence | Count  (30 seq. total) | Sequence | Count  (30 seq. total) |
| --- | --- | --- | --- |
| AREYGTRFSLIGGYR | 1 | PSVPVFRGRFFQVDY | 1 |
| AVVSSGGALYXRIVR | 1 | PVIAIPPSFANMFLF | 1 |
| FAFSPCPLESNVIGC | 1 | PWSSRPWYLQFLGAA | 1 |
| FSLGVSSVIFSPVSA | 1 | RLVCWRLGCVSPMGS | 1 |
| FVFPRPNAY* | 1 | RPPVVDAAHFGASRW | 1 |
| GHSPRCSSSFVRCEA | 1 | RQQDGRLIYTTASVR | 1 |
| GQDVRIRNEVNQYGL | 1 | SEELLVESSAIRSRE | 1 |
| GVQVPFSGGSSLLGM | 1 | SNPGLFVSGYWRLFP | 1 |
| GVSEDASVQHYYRSP | 1 | TRLECFSAGWRLSAC | 1 |
| LRMGLCSDQIRLSCA | 1 | TRVYWAPVSEGDVSP | 1 |
| MFRTFVGRSSNAVVG | 1 | VSSQHGXGREVNSAV | 1 |
| MQSVSGWFPWESVAY | 1 | VYASPASIPWSFAGL | 1 |
| NAVRVAFWSVPLYPF | 1 | YFTTPATLLPFGVGT | 1 |
| NAVRVAFWSVPLYXF | 1 | YRVREPQLFCCEGPD | 1 |
| PGGGANFLLSPFSGG | 1 | PSVPVFRGRFFQVDY | 1 |

**Table S2.** 54 sequences were randomly picked and identified from the 4^th^ round of *in vivo* biopanning.

| Sequence | Count  (54 seq. total) | Sequence | Count  (54 seq. total) |
| --- | --- | --- | --- |
| ACAIGCXQANGLALV | 1 | PFPVSSRVVPRFTAV | 1 |
| AGGGGPTVGDDRVRR | 1 | PIGVFWDDLLIRH | 1 |
| ALGRGRVVGVRLVSL | 1 | PKAFQYGGRAVGGLW | 3 |
| AREYGTRFSLIGGYR | 3 | PKAXQYGGRAVGGLW | 1 |
| AVYPLLVICLLGRFW | 1 | PVQSLYAIGGVSLDT | 1 |
| DGLRVGXWDAVSX | 1 | PWSSILVGRSSSLLS | 1 |
| DKCVRRXILFMLVLM | 1 | QDFFAFCGGLAAVCG | 1 |
| DVLYRFGHSSVVFPG | 1 | RCSSFAMCGSIVPGS | 1 |
| FPYTRVPHFGNVHSS | 1 | RFPSASWSFSGHAAT | 1 |
| GASILGGXXGXXP* | 1 | RGAPFFISVSERAFR | 1 |
| GLAPVGSHSATPRPW | 1 | RSSGAVXNGVGVSALG | 1 |
| GLDLLHTTWRCCAPP | 1 | SGTGLXYPSWTAGSS | 1 |
| GPVVLPWPLFDGHLS | 1 | SGVHSYLAXRPVRL | 1 |
| GRXRLGVVSVLSXDG | 1 | SIGEMPSGLVTLSST | 1 |
| GTLKVGMMCSLGACLG | 1 | SNPGLFVSGYWRLFP | 1 |
| HRWMPHVFAVRQGAS | 1 | SRHVRSLGNLGDVSVG | 1 |
| KTWPXLVPGSASGRA | 1 | TSASTSVFIXRASST | 1 |
| LDPRRASFHIGRAAP | 1 | TTCLDFFNRPEVFWG | 1 |
| LFTPFFSCHEFRCWD | 1 | TYHSVVWSEPVVWS | 1 |
| LGRAGQSYPSFARGL | 1 | VRCEGVLINGDRCGL | 1 |
| LGRARDRLSIQFPHF | 1 | VSSQHGXGREVNSAV | 1 |
| LRFISVAVAGNLSWA | 1 | WSNRMPPLFTXWYX | 1 |
| LRPPVAGMSVARVYG | 1 | YFTTPATLLPFGV* | 1 |
| LVRALPLWPLVGPV | 1 | PFPVSSRVVPRFTAV | 1 |
| MCAVRDPAFSRS* | 1 | PIGVFWDDLLIRH | 1 |
| MPLRFFXPSXGPLAP | 1 | PKAFQYGGRAVGGLW | 3 |
| PFARAPVEHHDVVGL | 1 | PKAXQYGGRAVGGLW | 1 |

**Table S3.** 162 sequences were randomly picked and identified from the 5^th^ round of *in vivo* biopanning.

| Sequence | Count  (162 seq. total) | Sequence | Count  (162 seq. total) |
| --- | --- | --- | --- |
| AHPPLASVWHVSVPL | 1 | PKAFQYGGRAVGGLW | 7 |
| ALESSGSVPSRDLPP | 1 | PLRYGPSRSRLDEVW | 1 |
| AREYGTRFSLIGGYR | 7 | PMLGPHLCVSEFCGR | 1 |
| ARIGFASGSRPYVSS | 1 | PNRNYLPNTSMDGSR | 1 |
| ASSFSVSEVSVVRRV | 1 | PPDSFSRGYRVRDTF | 1 |
| ASSVPYVDANWSYNR | 1 | PPQTISGKARYLPSV | 1 |
| AVFDGSLCVPGFYSC | 1 | PRAASIFRAGHVSML | 1 |
| AVTPQNPYGIQRDRR | 1 | PSALRVKGFTVVSRS | 1 |
| AYGLVVPDLPAMPSL | 1 | PVRYGFSGPRLAELW | 3 |
| CAGFQSRQVAL* | 1 | QADGPNSVVRPFTLT | 2 |
| CSGCAPGFRSERAIR | 1 | QPVSAVPLCRLHCRS | 1 |
| DRYLPINGVSMFGVP | 2 | QPWHPGVYGVASSVA | 1 |
| DSALRVSRWRLSHSV | 1 | RAPRSAPGIFVFRSF | 1 |
| DSSSLGNSSGSRGWR | 1 | RERIHSPGSTQILFL | 1 |
| EGLFSFPRGASE* | 1 | RFGFSATWDQSNLLL | 1 |
| FAFSPCPLESNVIGC | 1 | RGFLSDLHALASRDR | 1 |
| FGHIIPSRFDRLSLG | 1 | RNVPPIFKEVYWIAQ | 3 |
| FGRIPSPLAYTYSFR | 1 | RRVP* | 1 |
| FLLTGLQHATSSGFR | 1 | RSDHLGVGPASASRYG | 1 |
| FSDSFVTGVWAPSRP | 1 | RSFAYAAAPTSFPWV | 2 |
| FSSGGTSYRLRHIPF | 1 | RSLSHGGRWGPGYAI | 1 |
| FSVSFPSLPAPPDRS | 1 | RSPWSDFYASASRGP | 1 |
| FVFPRPNAY* | 1 | RTLIRMGTGAHAFAV | 3 |
| FVRHYLLGGQGSLPR | 1 | RVLFLVAALAIGSLA | 1 |
| GAKPGLGPRAHLGGV | 1 | RVLGRDGSVFYELAA | 1 |
| GALHQTRPVGPAFAW | 1 | RVNFLFKPSVIFNAP | 1 |
| GAPGAFFGSVRDVVPRR | 1 | RVPPRYHAKISPMVK | 1 |
| GAPLSANFNNPAFWR | 1 | RVQSTILSGLRGFSS | 1 |
| GAVDFVSLYAAATVA | 1 | RVTHHAFLGAHRTVG | 1 |
| GAYPSLDFPRAPSFG | 1 | RYRPVFPGFEETLPR | 1 |
| GDAFGFFPPFRTMG | 1 | SDHSPTQSQRASHDA | 1 |
| GFTDVHLHLPGNSHR | 1 | SDWFPTACFDCRSVR | 1 |
| GGAVFPEYPLARAFL | 1 | SGASRNSAFWAVSVA | 1 |
| GGPLVSPLSEDARPW | 1 | SGAYSSFRPSHHTTR | 1 |
| GKGEVSLYSLGSPGM | 1 | SGLCRYESPSGRPSC | 1 |
| GLHGATPAHRLFHTG | 1 | SGNSAIWPRVRLLHG | 1 |
| GSGAVSPPLWAARGR | 1 | SGYMTSALIRPSRFP | 1 |
| GSGTAYMMRPSLGPD | 1 | SKKPQSSNEGYRIAD | 1 |
| GTRAVLVVGLDALSA | 1 | SKTFQYGGRAIEGPW | 1 |
| GVGHVRAGHLRSVGI | 1 | SLPHLAPFGTTFFGP | 1 |
| GWSHFYRSPNFLRIQ | 1 | SPPLAPYGGTRVGLT | 1 |
| HAALSLPWYRINSVY | 1 | SPPLAPYGGTRVGLTG | 1 |
| HGSLGLGWPGHTSVR | 1 | SSGFRDAFRGWDGSA | 1 |
| HKAIQHGGRAVGGAW | 1 | SSVA* | 1 |
| HPTTSYSTSVFSWGW | 1 | STFFGFVGNSIVRPW | 1 |
| HVSGYVSRFDRSVGA | 1 | STSGVLSSILHVVSV | 1 |
| HVTCVHSVSSLRSIV | 1 | SVGCPVVGTVGYLRCG | 2 |
| IPLLVNLPHLPRAAL | 1 | SVLHPALDFTSLA | 1 |
| IPVQFSTIDFVAASY | 2 | SVVXLRTRHFSTDSA | 1 |
| KVGVALVGP* | 1 | TGSVLSRFASGRLAP | 1 |
| LAGVQMSLRSLDTRR | 1 | TGTVVQVADPFAGGH | 1 |
| LFLNSHDDDRSFLSS | 1 | TNVPPISNDA* | 1 |
| LGAHVVLSGSSDFFP | 1 | TPFVFRAGRFFLHAG | 1 |
| LGGAGPFFGLGLVES | 1 | TRLECFSAGWRLSAC | 1 |
| LLGELPPTPRSPRLW | 1 | VCSPVFSPFCKMSVA | 1 |
| LPSIGPWEPXPDALS | 1 | VFGATSVVRDLYSLR | 1 |
| LRPPVAGMSVARVYG | 1 | VFRRVDTAVYKPSYP | 1 |
| LRSLVSYSGGHNYSG | 1 | VGAASFYLERGSRXS | 1 |
| LTLSHPHWVLNHFVS | 1 | VLERSRRVLGGAAKV | 1 |
| LVGTLLGAHGFVAIP | 1 | VRINNCIGFDSNCTS | 1 |
| LVSRLTPCDLSFYAC | 1 | VRMFDYGVPRRAVYG | 2 |
| MAAWLPHSYSITRLL | 1 | VRVTPSFFREPSGFV | 1 |
| MHFGPGFGH* | 5 | VTATAAQPGDAFIGV | 1 |
| NAAGYNSRVVTLPLF | 1 | WFPTHPFWTYSSWTG | 1 |
| PAQSNFVTWGYNVAV | 1 | WRYRLVYALLAMLTI | 1 |
| PFARAPVEHHDVVGL | 1 | YSRVLSSSSYRFFDR | 1 |
| PGHSLGKLSVLHSFF | 2 |  |  |

The * marker indicates the incomplete peptides displayed on particular phages, which might be the result of either the failure of sequencing or the insertion of a stop codon into the fusion DNA sequence.


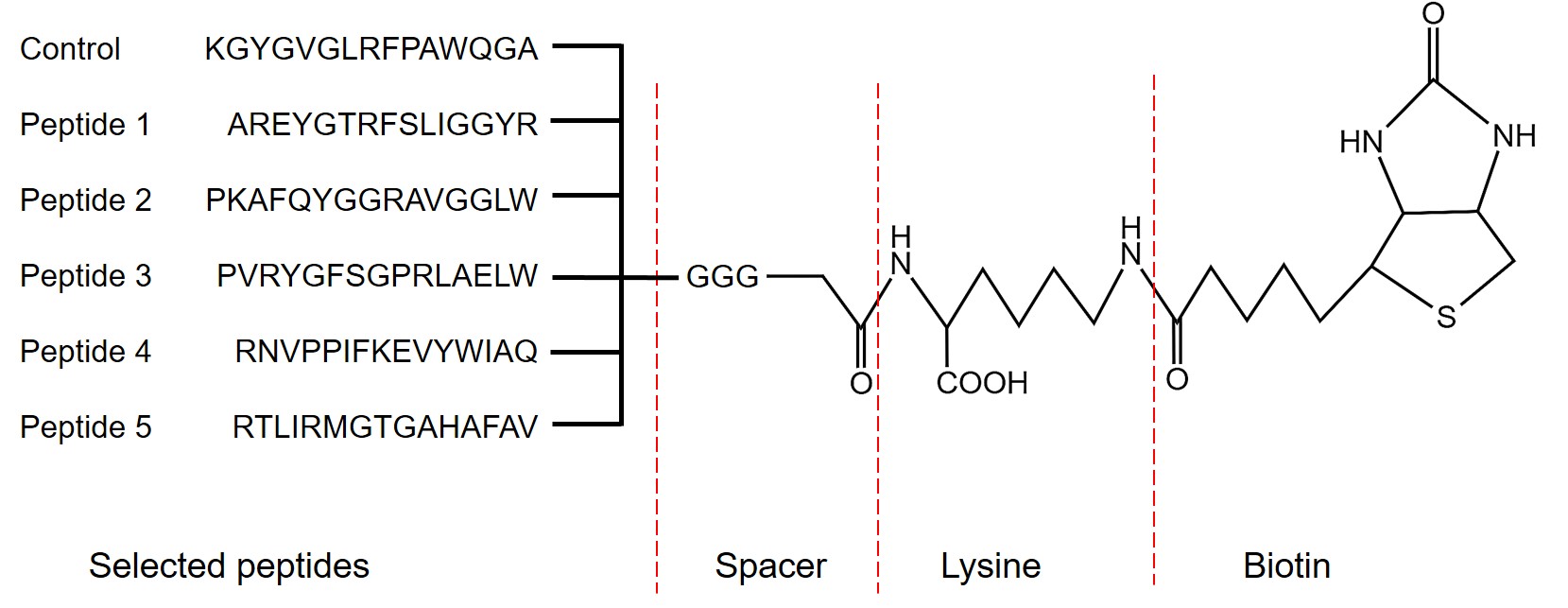


**Figure S1.** The general structure of the biotin labeled peptides is: N' selected peptide-GGG-K-biotin C’. For example, for peptide 1, the biotinylated structure is N' AREYGTRFSLIGGYR -GGG-K-biotin C'. The biotin modification was conducted at C-terminus, where an extra lysine (K) was added to allow the formation of amide bond between its side chain amine group and the carboxyl group of biotin. A commonly used spacer, GGG, was added between the selected peptides and biotin to protect the secondary structure and the functionality of the peptides.


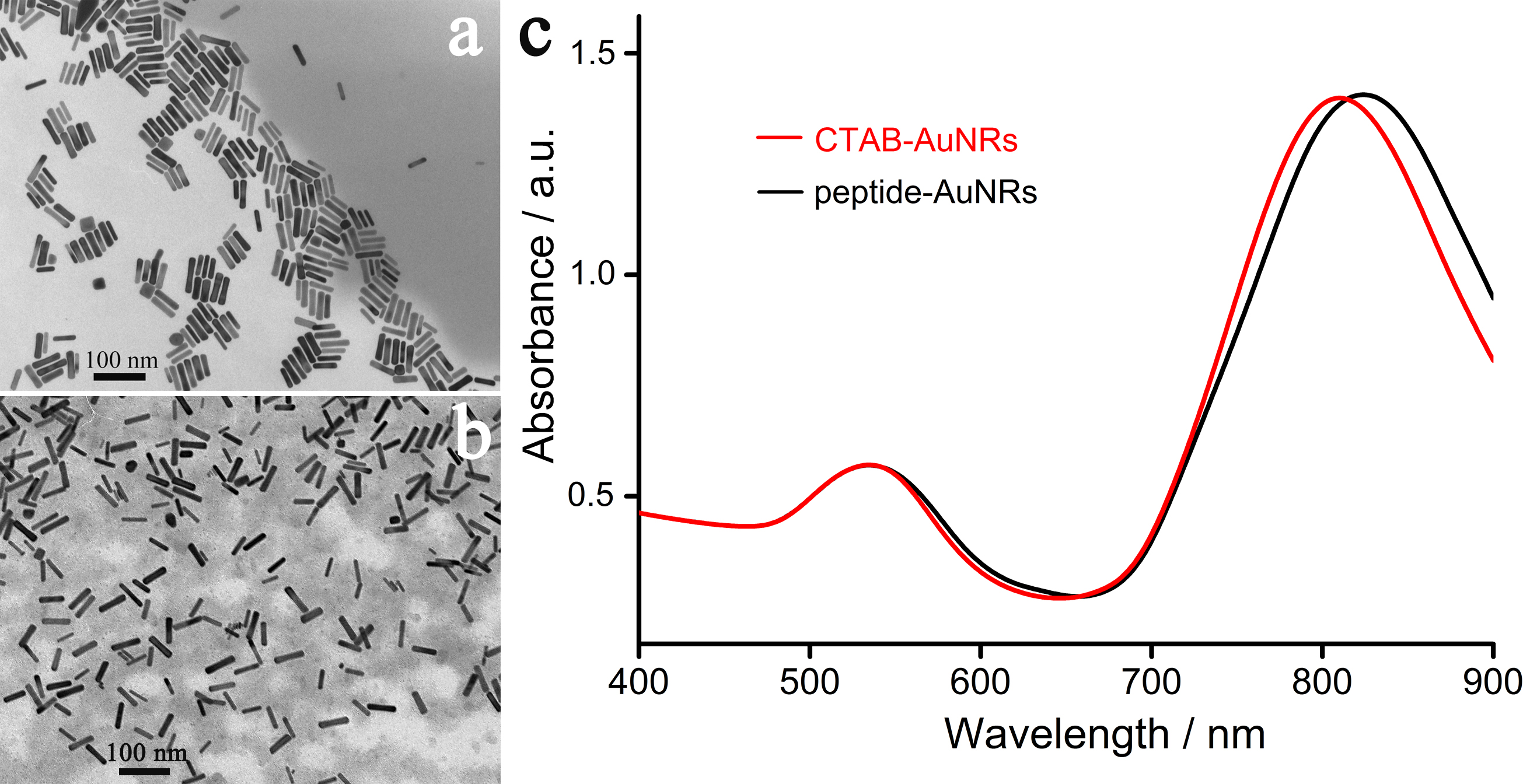


**Figure S2.** TEM images and UV-Vis spectra of the as-synthesized CTAB-AuNRs (a) and peptide functionalized AuNRs (b). No apparent AuNRs aggregation was observed after peptide modification. The localized surface plasmon resonance (LSPR) peak was shifted from 810 nm to 821 nm as a result of peptide functionalization. AuNRs in b were negatively stained, however, the peptides layer was not visible.


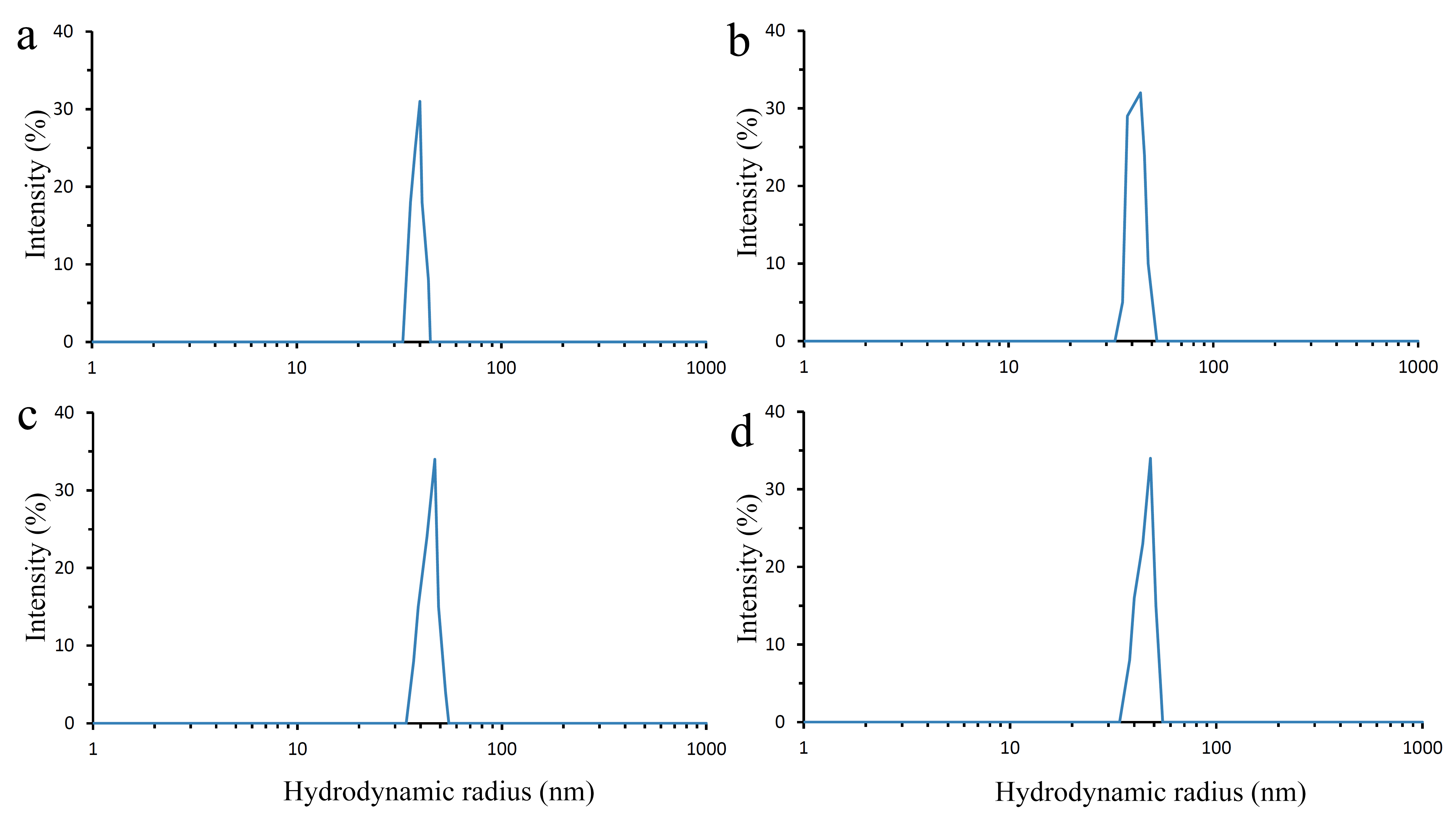


**Figure S3.** Dynamic Light Scattering (DLS) measurement of AuNRs with different surface ligands. The effective hydrodynamic radii are (a) 40.8 ± 2.1 nm for the as-synthesized CTAB-AuNRs in water, (b) 44.3 ± 2.5 nm for the PEG functionalized AuNRs in water, (c) 47.0 ± 2.4 nm for the peptide functionalized AuNRs in water, and (d) 47.6 ± 2.7 nm for the peptide functionalized AuNRs in PBS.

**Figure S4.** Blood circulation of AuNRs. 1.0 mg of tumor targeting peptide conjugated AuNRs (green) or control peptide conjugated AuNRs (blue) were injected intravenously. To track the gold concentration in peripheral blood (represented as the mass of gold per ml of blood), 20 µl of blood was collected from suborbital space of the mouse each time at 3 min, 20 min, 1 h , 4 h, 7 h, 12 h, 24 h and 48 h after injection, and immediately added into 200 µl of freshly prepared aqua regia. The concentration of AuNRs was quantified by ICP-AES.


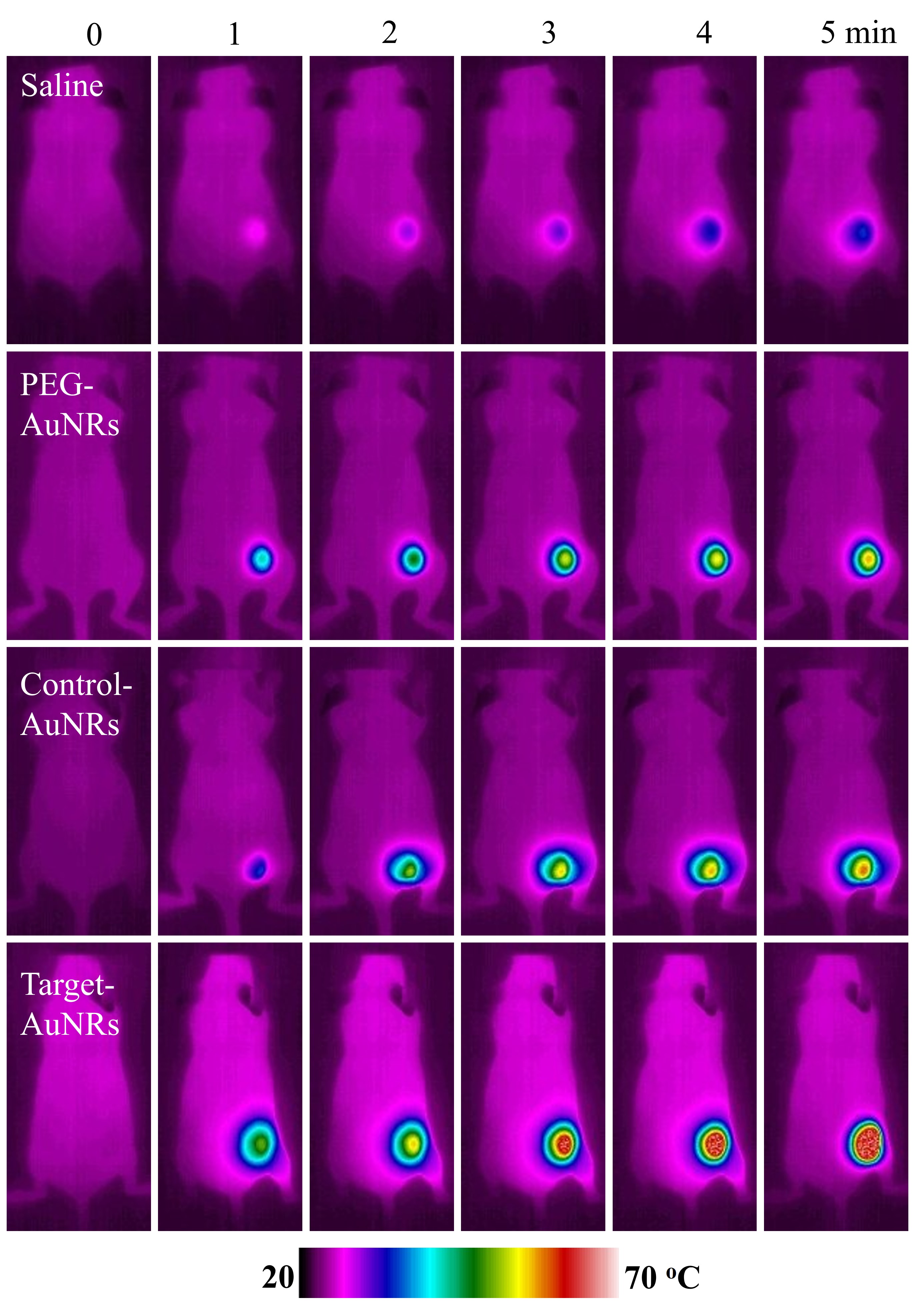


**Figure S5.** Thermal imaging of temperature increase in tumors during the photothermal treatment.


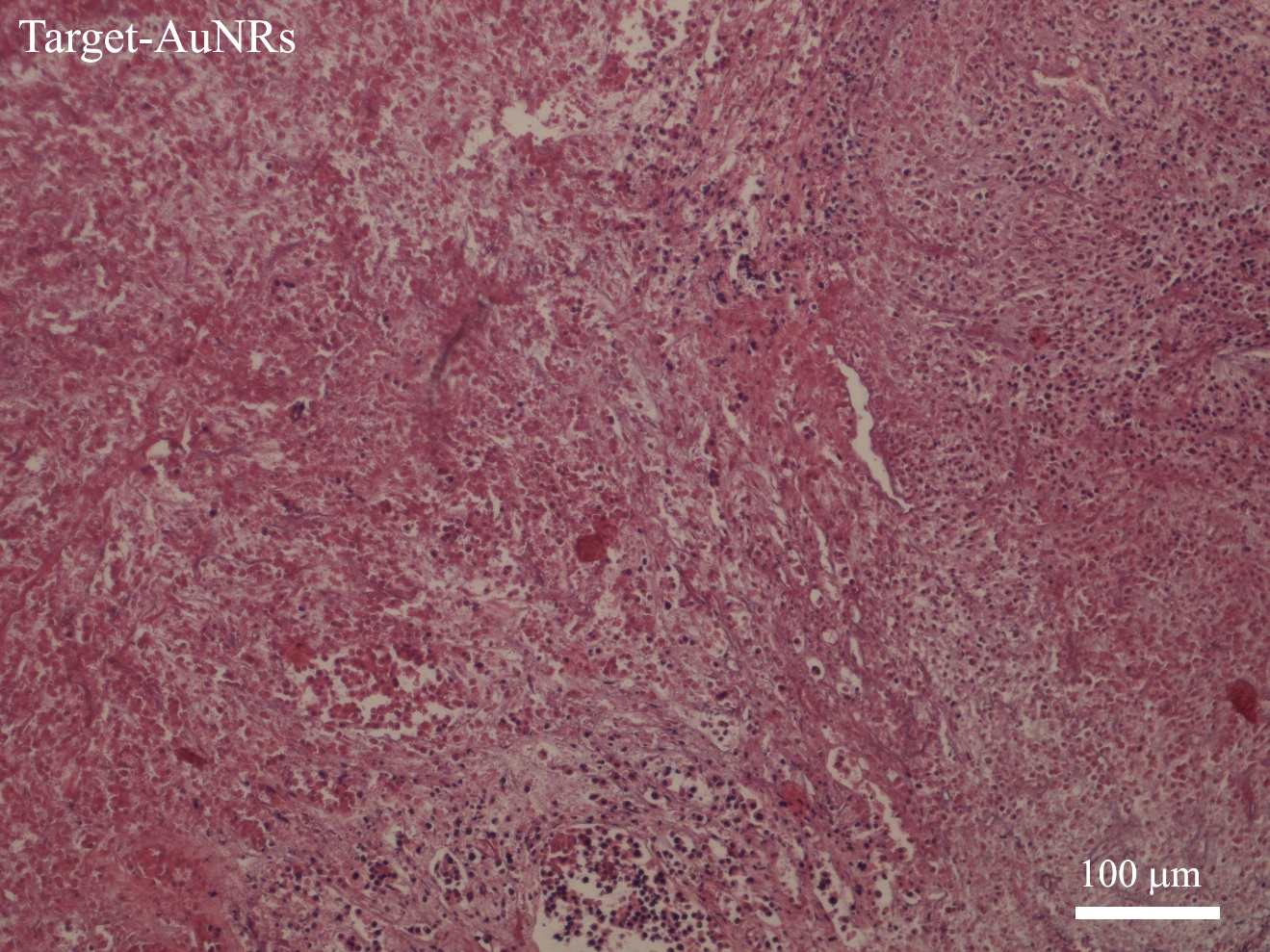


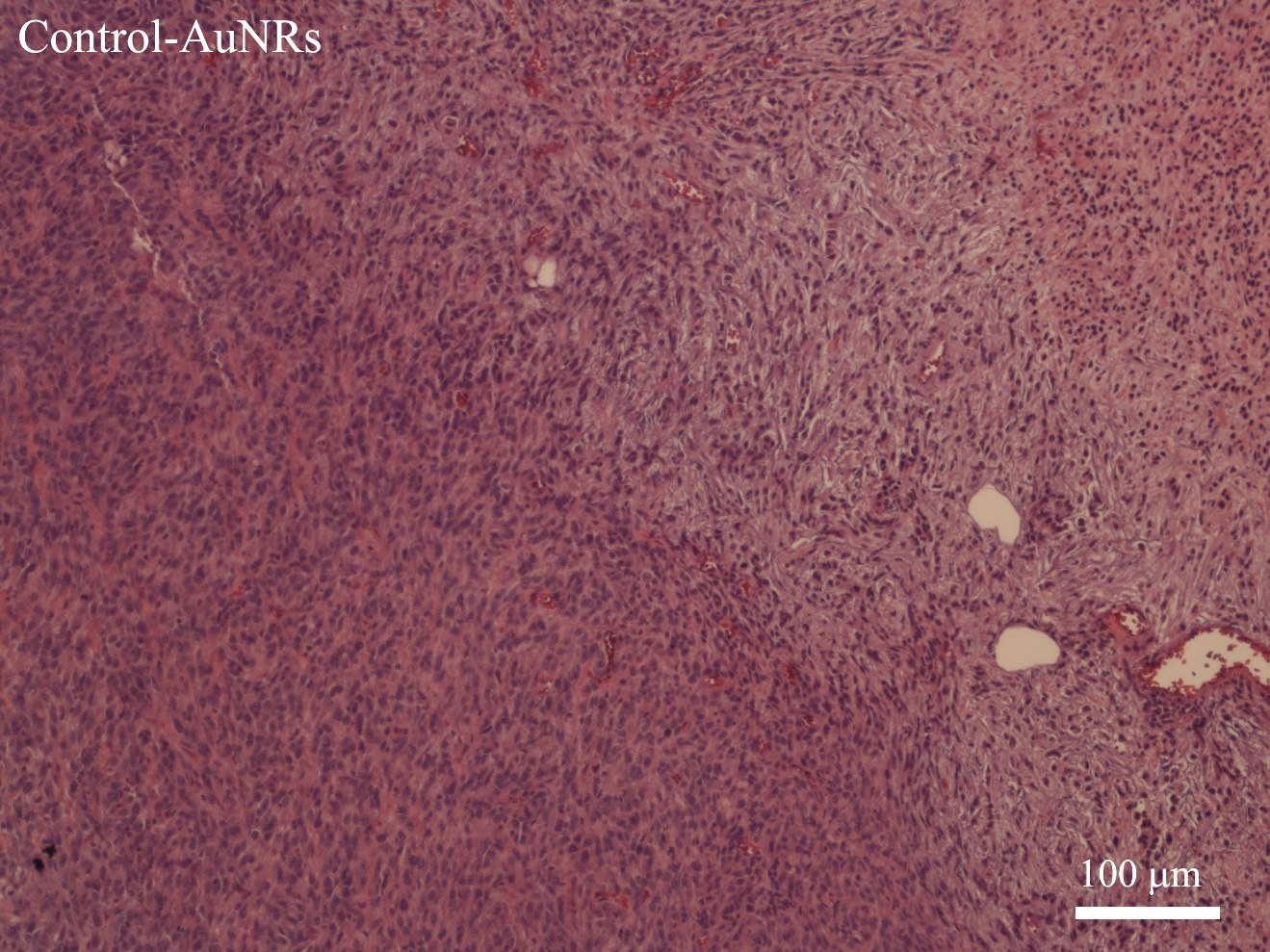


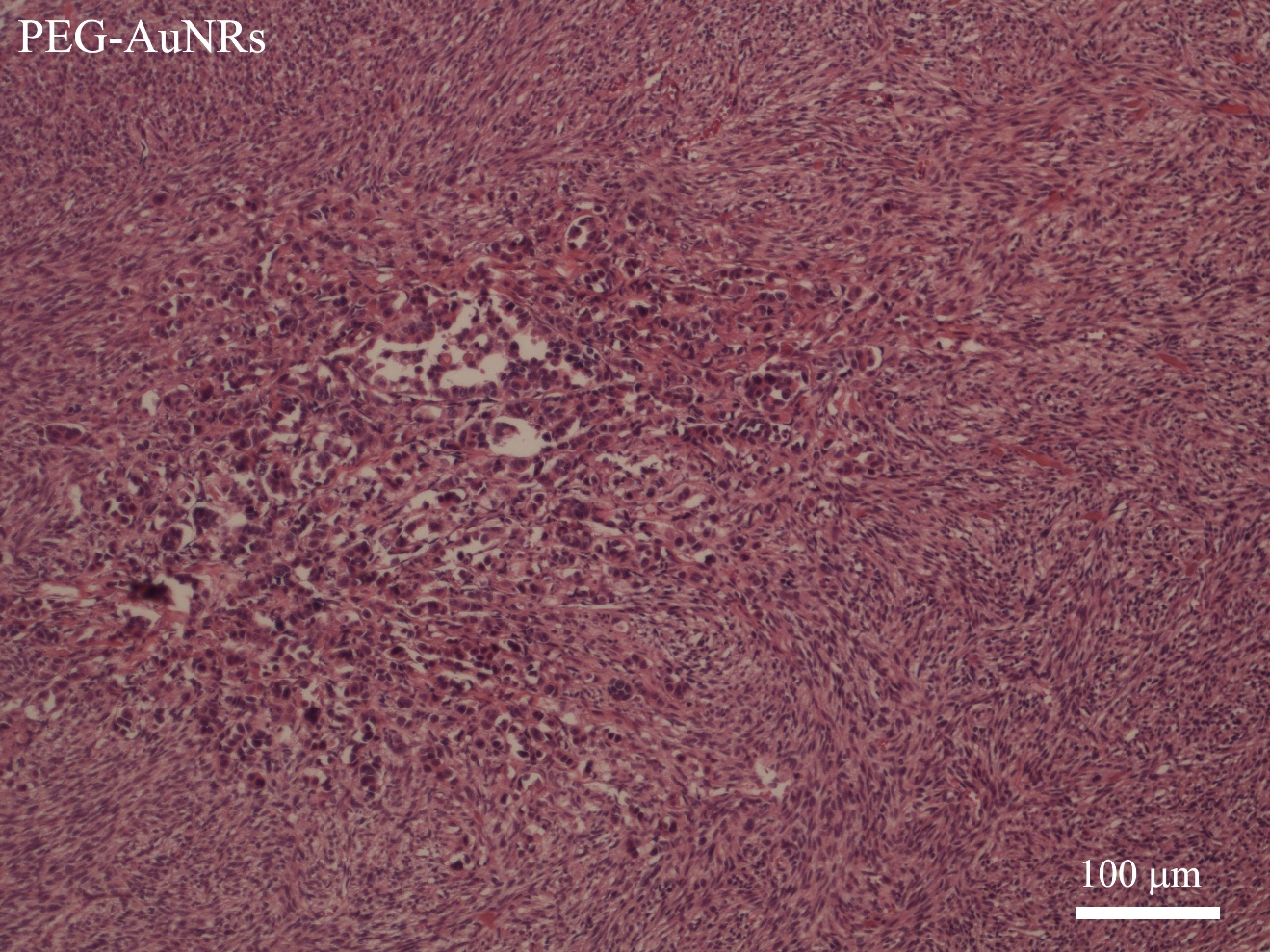


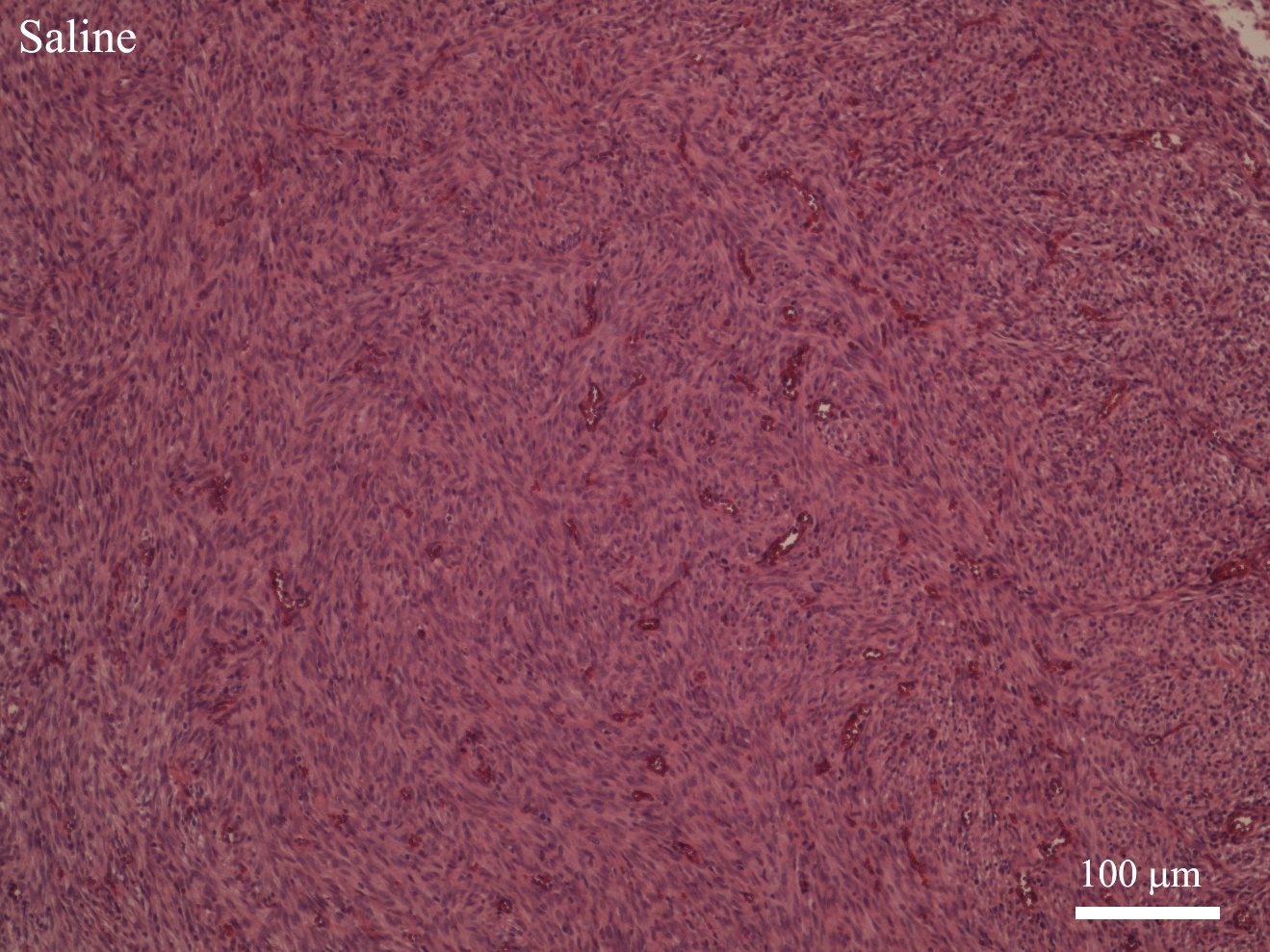


**Figure S6.** Low magnification optical images of HE stained tumor sections.
